# Supplementary material for: HPLC-PDA Method for Quantification of Bioactive Compounds in Crude Extract and Fractions of Aucklandia costus Falc. and Cytotoxicity Studies against Cancer Cells
Source: Molecules. 2023 Jun 16;28(12):4815. doi: 10.3390/molecules28124815 (PMC10303526; doi:10.3390/molecules28124815)
Supplement: Supplementary file 1 [file molecules-28-04815-s001.zip › molecules-2203020-SI.pdf]

Table of contents

Fig S1: ^{1}H NMR spectra of compound Dehydrocostus lactone.
Fig S2: ^{13}C NMR spectra of compound Dehydrocostus lactone
Fig S3: DEPT spectra of compound Dehydrocostus lactone.
Fig S4: Purity profile of compound Dehydrocostus lactone.
Fig S5: HRMS of compound Dehydrocostus lactone
Fig S6: ^{1}H NMR spectra of compound Costunolide.
Fig S7: ^{13}C NMR spectra of compound Costunolide.
Fig S8: DEPT spectra of compound Costunolide.
Fig S9: Purity profile of compound Costunolide.
Fig S10: HRMS of compound Dehydrocostus lactone
Fig S11: ^{1}H NMR spectra of compound Syringin.
Fig S12: ^{13}C NMR spectra of compound Syringin.
Fig S13: DEPT spectra of compound Syringin.
Fig S14: Purity profile of compound Syringin.
Fig S15: HRMS of compound Syringin.
Fig S16: ^{1}H NMR spectra of compound 5-Hydroxymethyl-2-furaldehyde
Fig S17: ^{13}C NMR spectra of compound 5-Hydroxymethyl-2-furaldehyde.
Fig S18: DEPT spectra of compound 5-Hydroxymethyl-2-furaldehyde.
Fig S19: Purity profile of compound 5-Hydroxymethyl-2-furaldehyde.
Fig S20: HRMS of compound 5-Hydroxymethyl-2-furaldehyde.
Fig S21: Calibration plot of compound Dehydrocostus lactone.
Fig S22: Calibration plot of compound Costunolide.
Fig S23: Calibration plot of compound Syringin.
Fig S24: Calibration plot of compound 5-Hydroxymethyl-2-furaldehyde.
Fig S25: HPLC Chromatogram of Hexane fraction.
Fig S26: HPLC Chromatogram of Chloroform fraction.
Fig S27: HPLC Chromatogram of Butanol fraction.

Compound 1: Dehydrocostus lactone

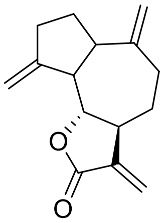

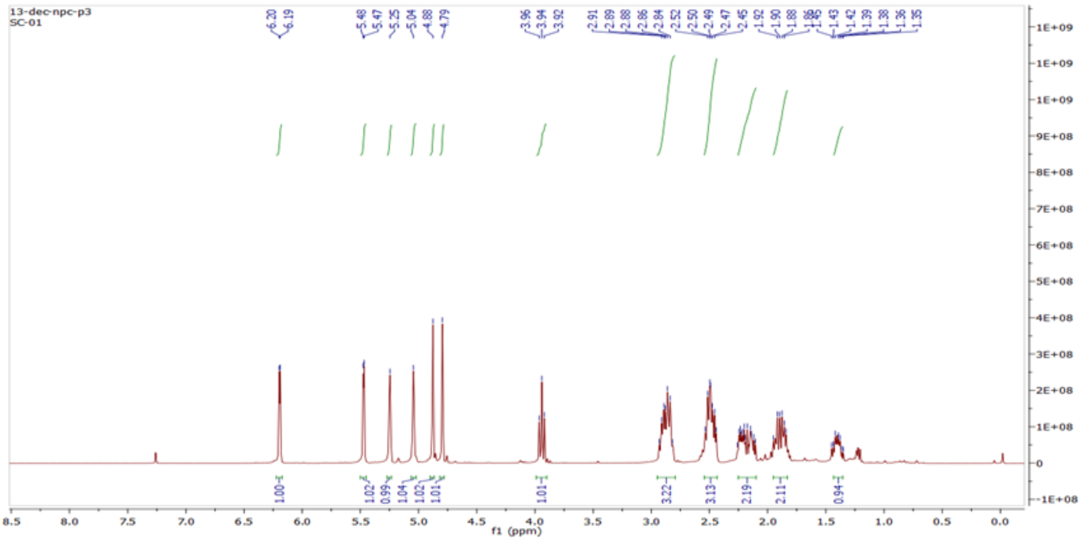

Figure S1: ^{1}H NMR spectra of Dehydrocostus lactone ($CDCl_3$, 400 MHz).

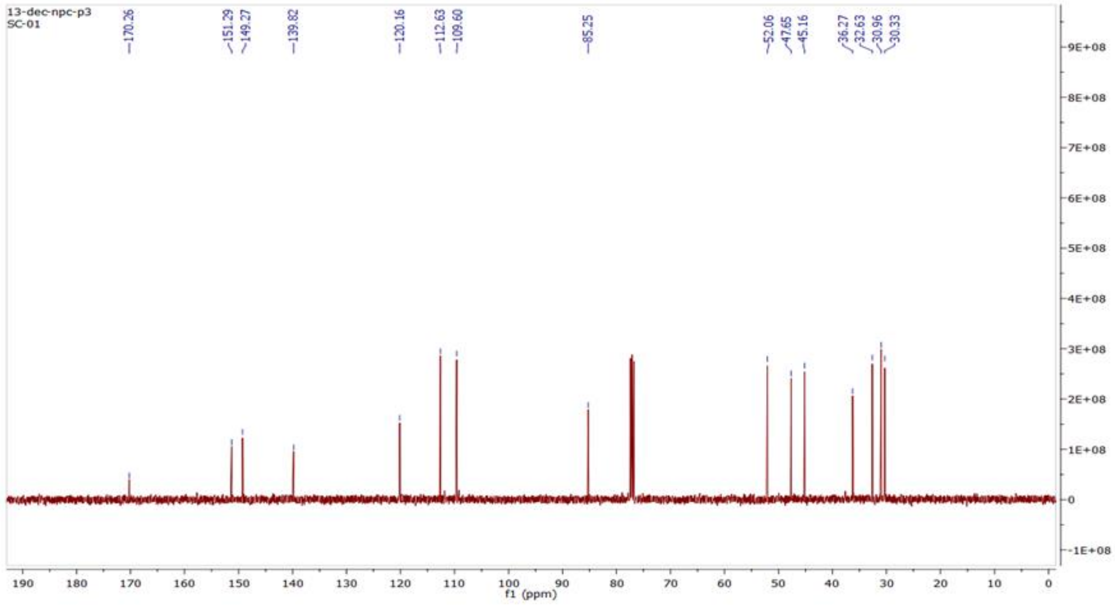

Figure S2: ^{13}C NMR spectra of Dehydrocostus lactone ($CDCl_3$, 100 MHz).

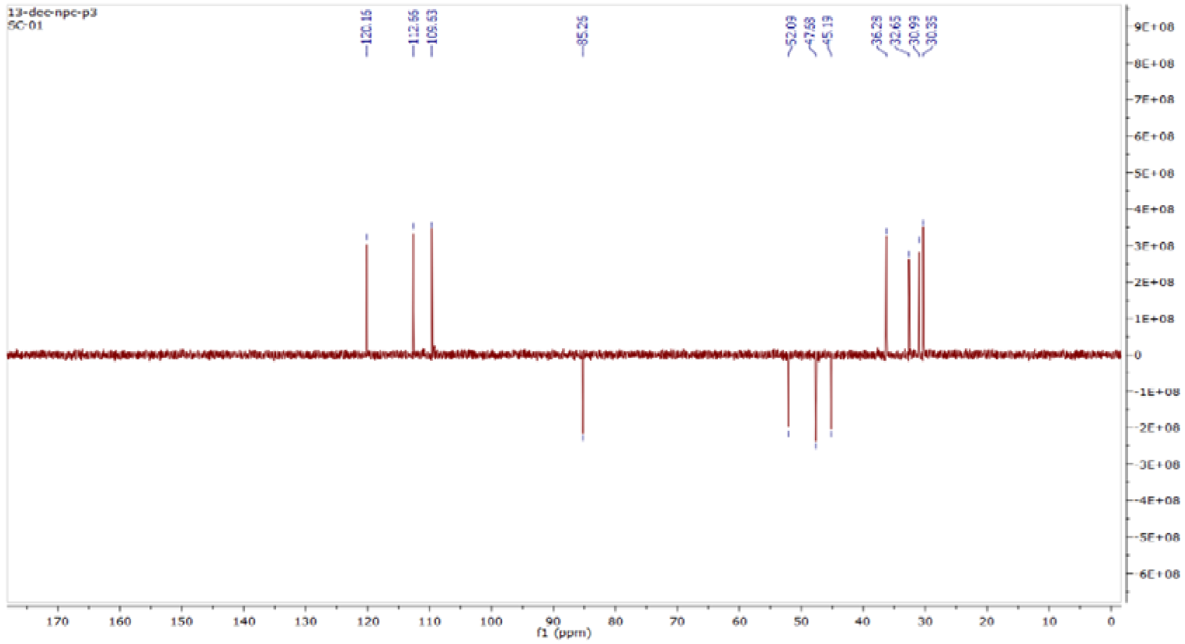

Figure S3: DEPT spectra of Dehydrocostus lactone ($CDCl_3$, 400 MHz).

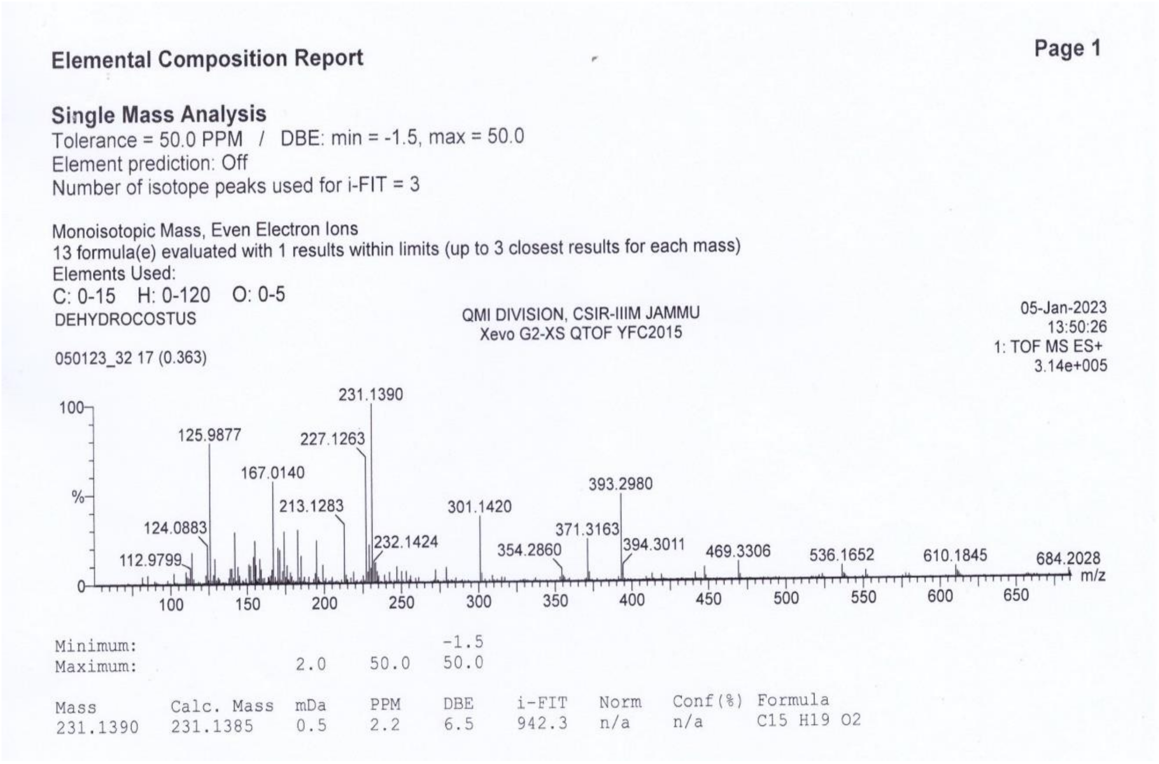

Figure S4: HRMS of Dehydrocostus lactone ($CDCl_3$, 400 MHz).

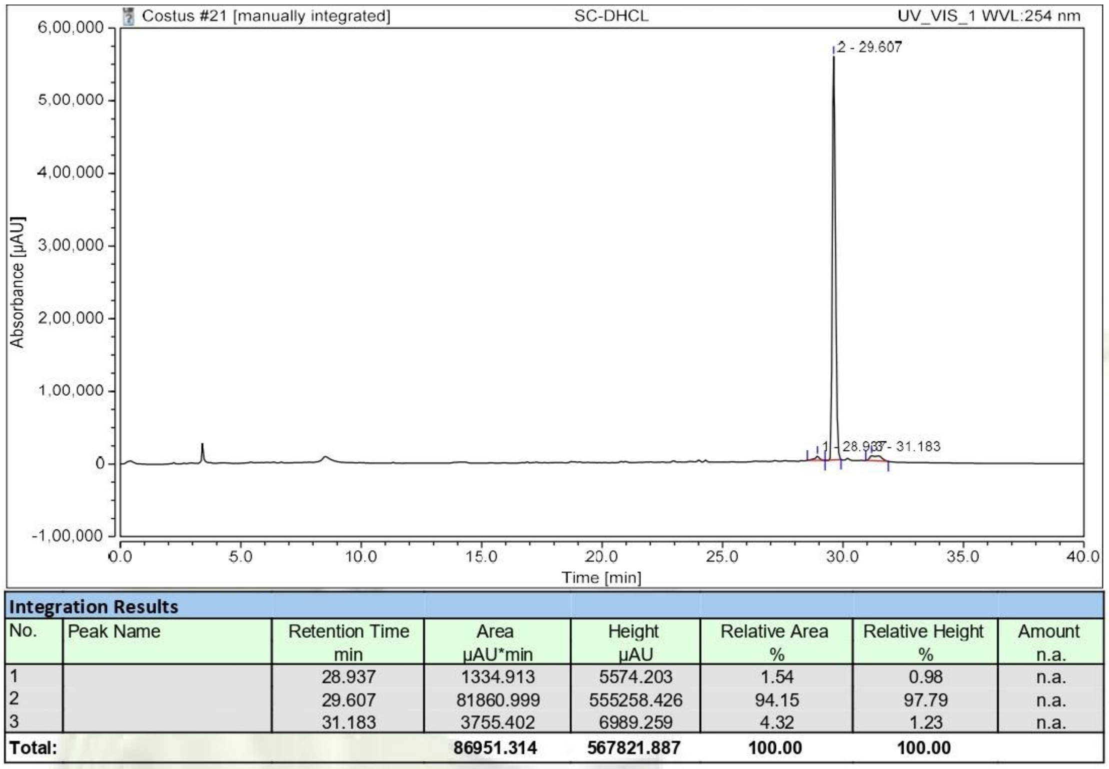

Integration Results							
No.	Peak Name	Retention Time min	Area µAU*min	Height µAU	Relative Area %	Relative Height %	Amount n.a.
1		28.937	1334.913	5574.203	1.54	0.98	n.a.
2		29.607	81860.999	555258.426	94.15	97.79	n.a.
3		31.183	3755.402	6989.259	4.32	1.23	n.a.
**Total:**			**86951.314**	**567821.887**	**100.00**	**100.00**	

Fig S5: Purity profile of compound Dehydrocostus lactone.

Compound 2: Costunolide

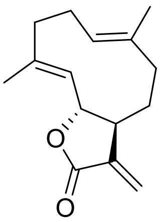

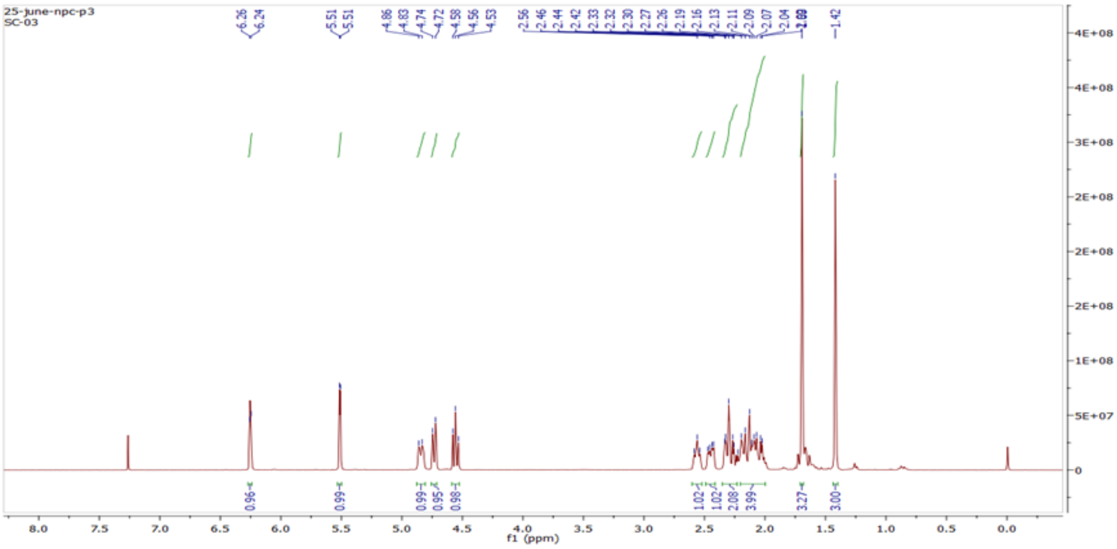

Figure S6: ^{1}H NMR spectra of Costunolide ($CDCl_3$, 400 MHz).

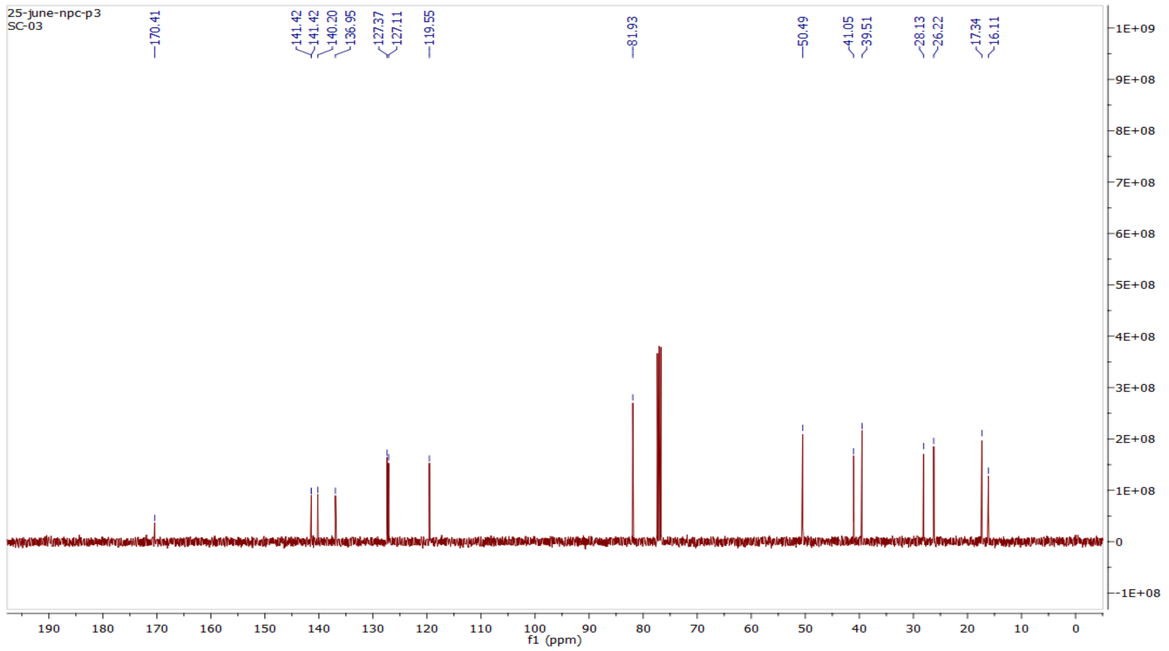

Figure S7: ^{13}C NMR spectra of Costunolide ($CDCl_3$, 100 MHz).

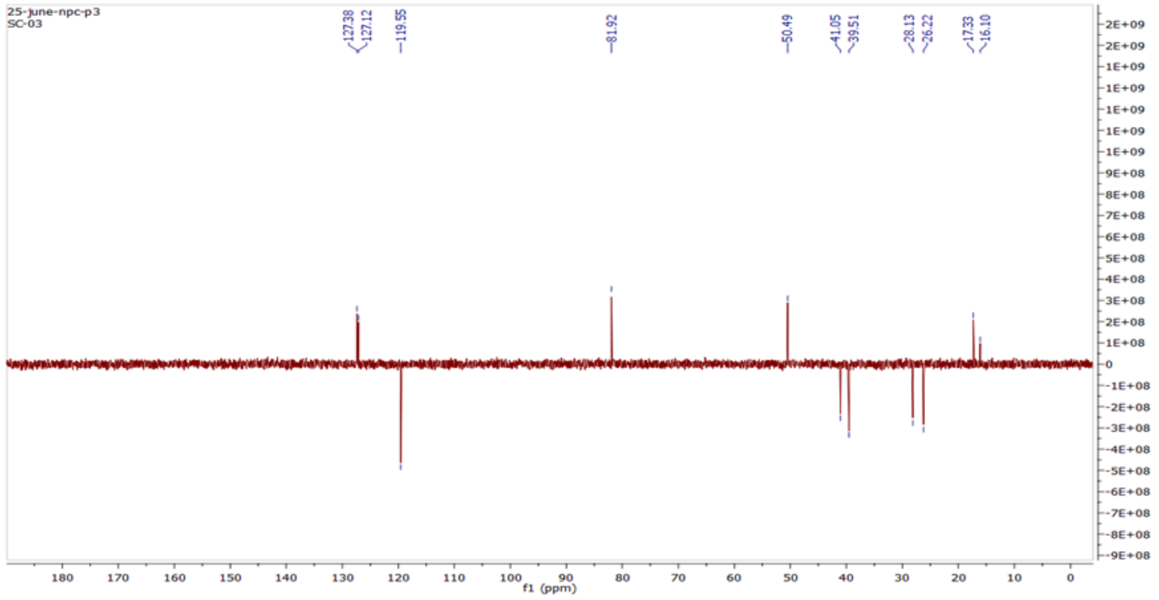

Figure S8: DEPT spectra of Costunolide ($CDCl_3$, 400 MHz).

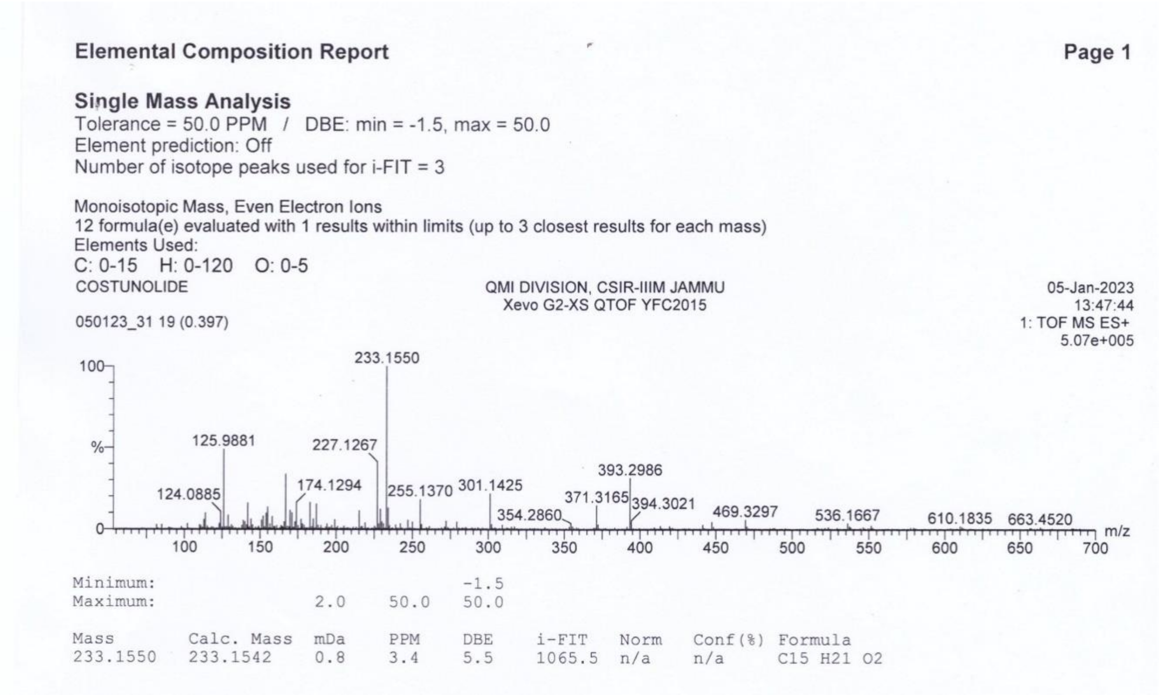

Figure S9: HRMS of Costunolide ($CDCl_3$, 400 MHz).

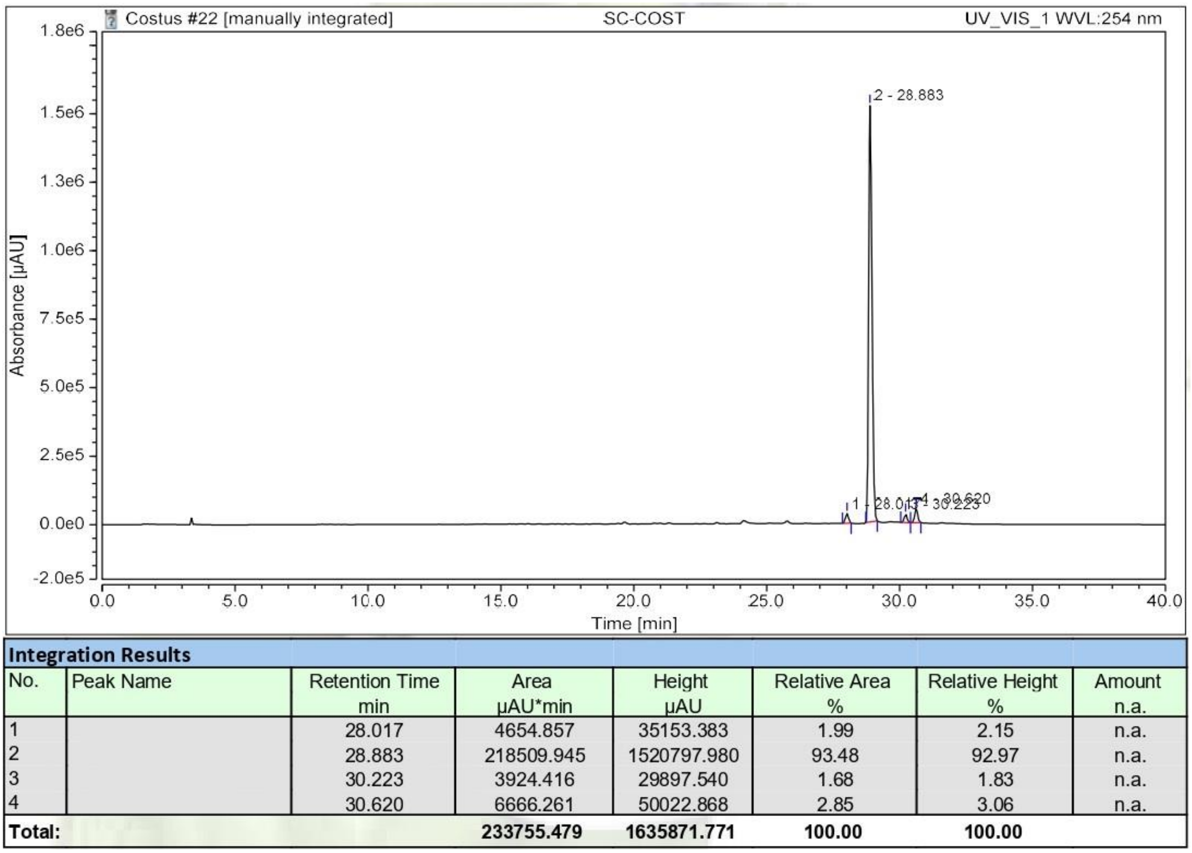

Integration Results							
No.	Peak Name	Retention Time min	Area µAU*min	Height µAU	Relative Area %	Relative Height %	Amount n.a.
1		28.017	4654.857	35153.383	1.99	2.15	n.a.
2		28.883	218509.945	1520797.980	93.48	92.97	n.a.
3		30.223	3924.416	29897.540	1.68	1.83	n.a.
4		30.620	6666.261	50022.868	2.85	3.06	n.a.
**Total:**			**233755.479**	**1635871.771**	**100.00**	**100.00**	

Fig S10: Purity profile of compound Costunolide.

Compound 3: Syringin

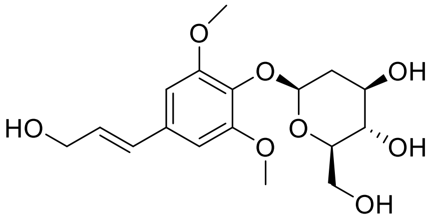

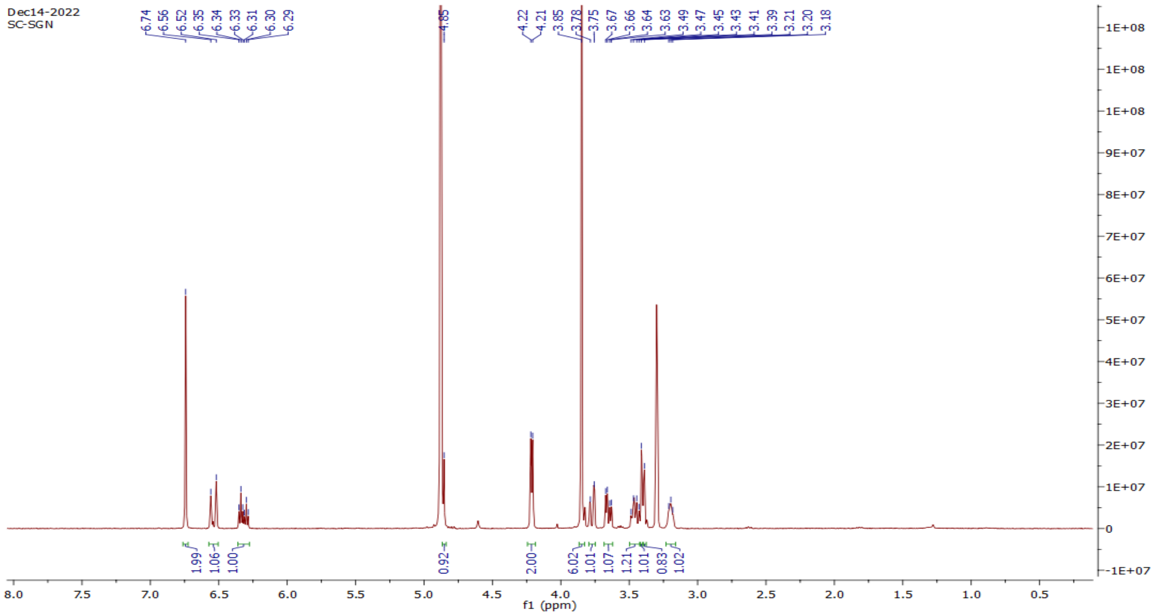

Figure S11: ^{1}H NMR spectra of Syringin ($CDCl_3$, 400 MHz).

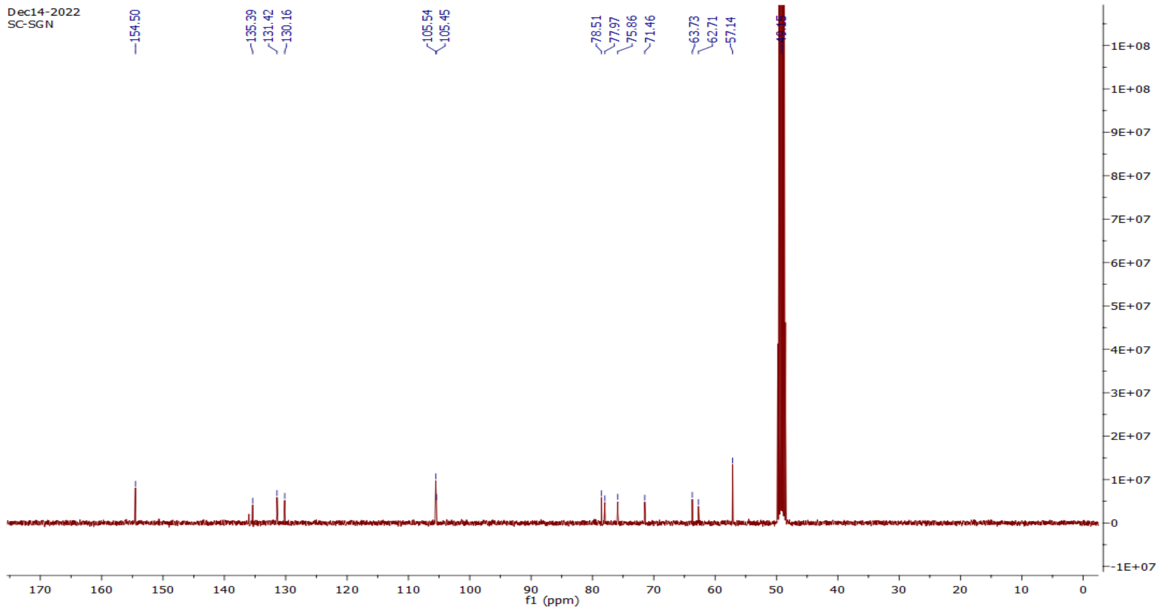

Figure S12: ^{13}C NMR spectra of Syringin (CD_3OD, 100 MHz).

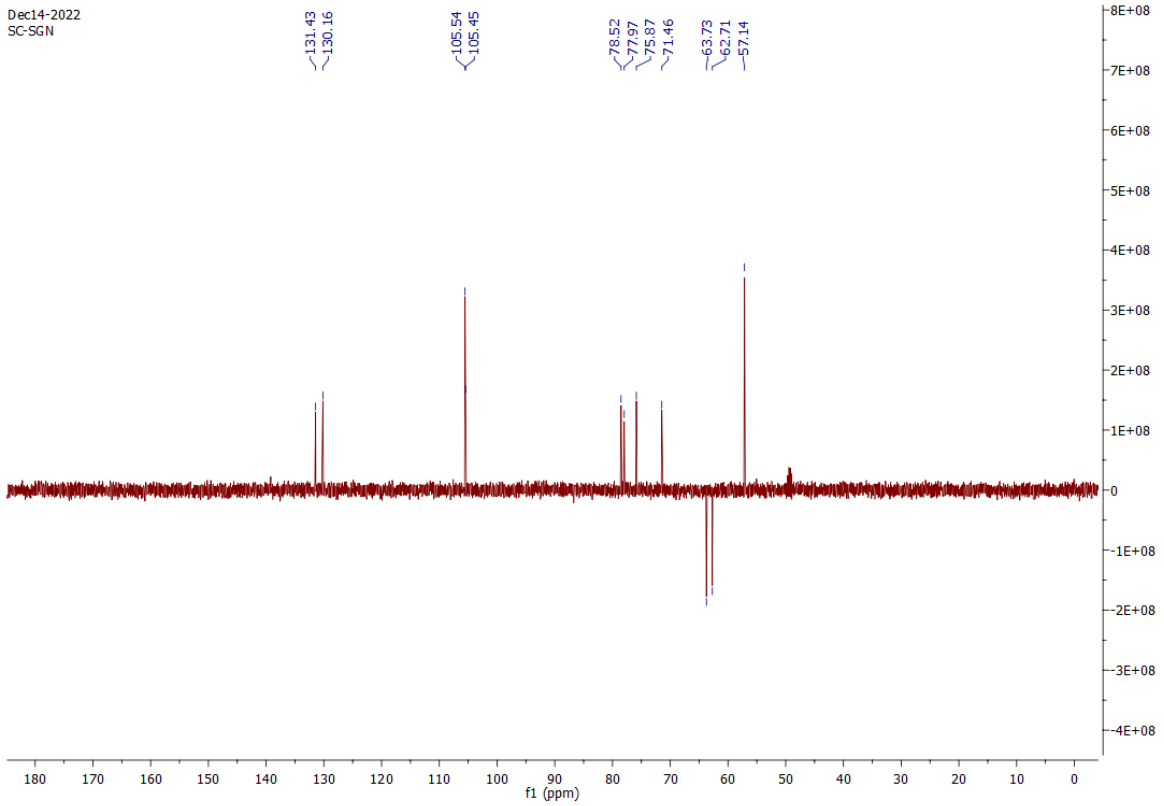

Figure S13: DEPT spectra of Syringin (CD_3OD, 400 MHz).

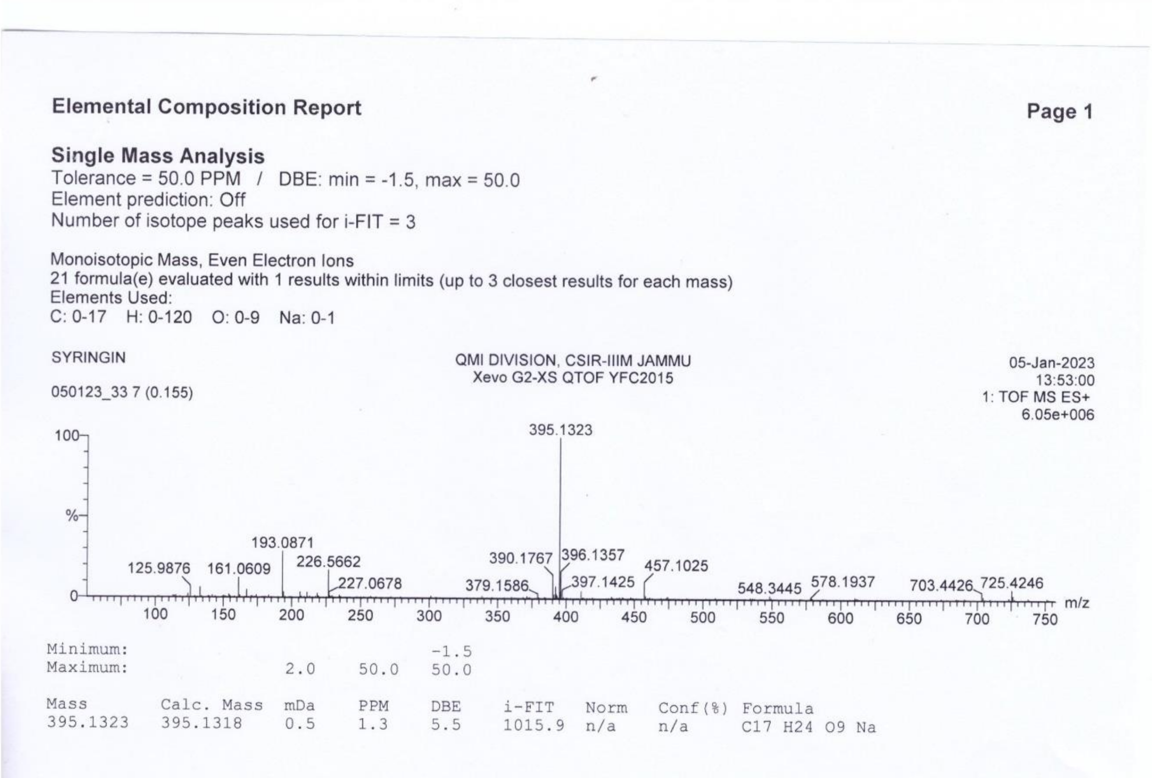

Figure S14: HRMS of Syringin (CD_3OD, 400 MHz).

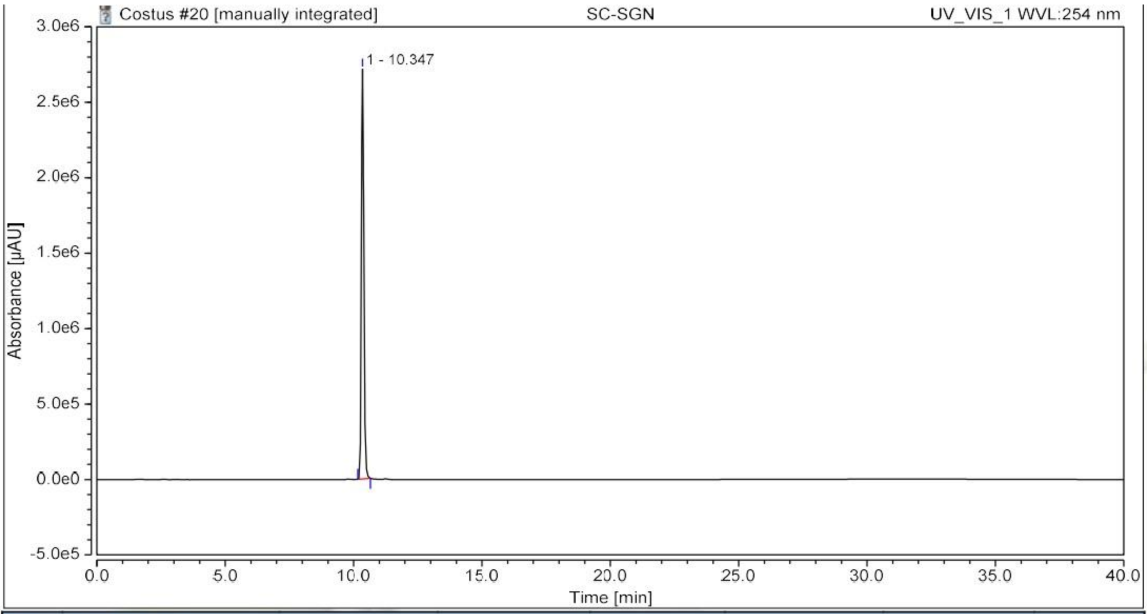

Integration Results							
No.	Peak Name	Retention Time min	Area µAU*min	Height µAU	Relative Area %	Relative Height %	Amount n.a.
1		10.347	297850.249	2716198.622	100.00	100.00	n.a.
**Total:**			**297850.249**	**2716198.622**	**100.00**	**100.00**	

Fig S15: Purity profile of compound Syringin.

Compound 4: 5-Hydroxymethyl-2-furaldehyde

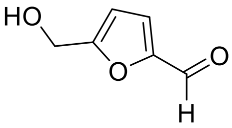

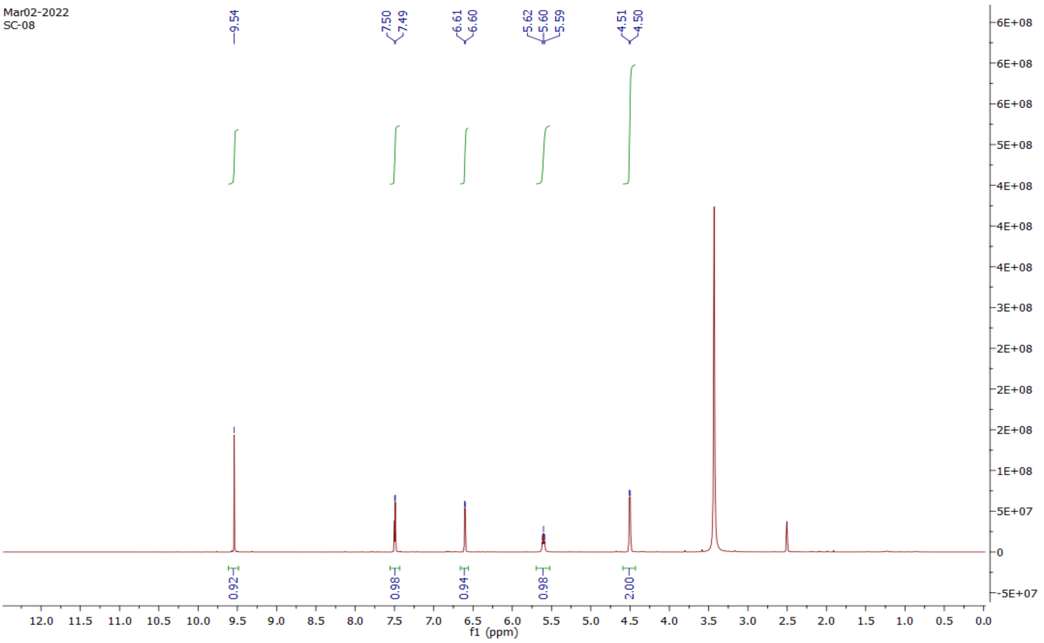

Figure S16: ^{1}H NMR spectra of 5-Hydroxymethyl-2-furaldehyde (DMSO d_6, 400 MHz).

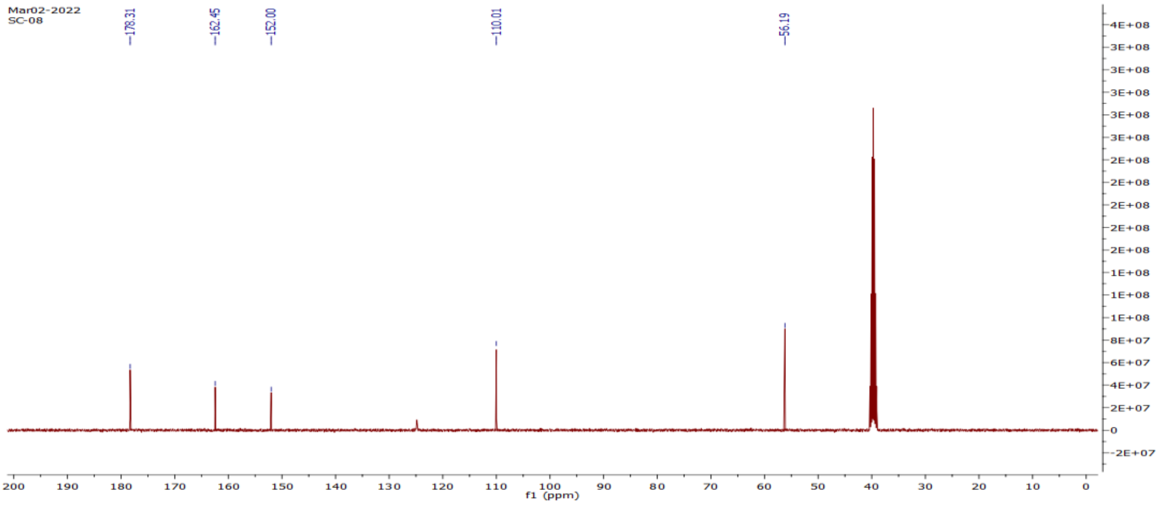

Figure S17: ^{13}C NMR spectra of 5-Hydroxymethyl-2-furaldehyde (DMSO d_6, 100 MHz).

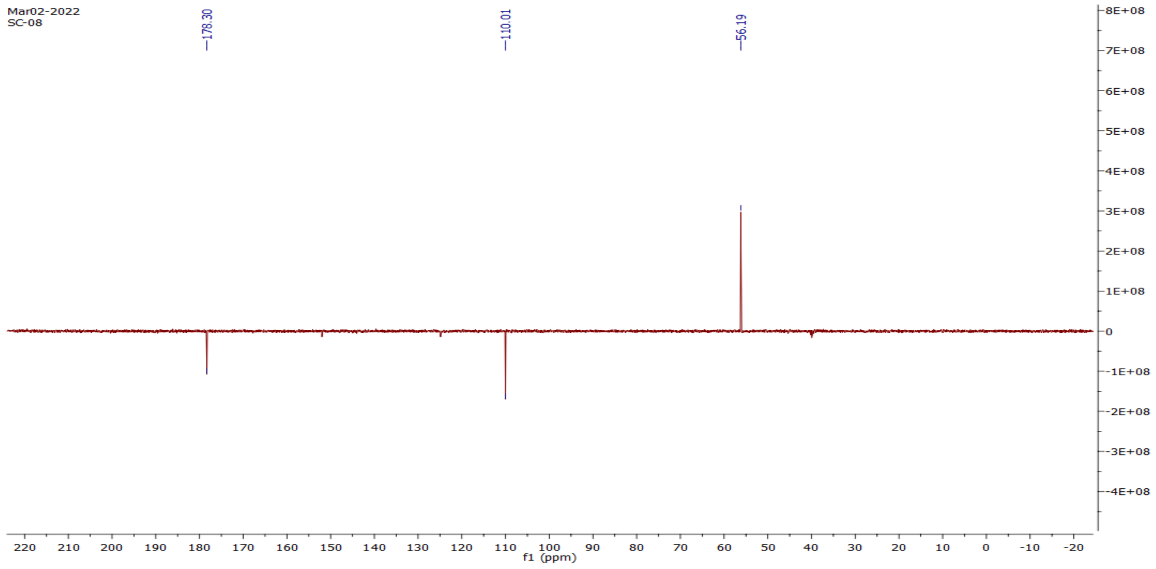

Figure S18: DEPT spectra of 5-Hydroxymethyl-2-furaldehyde (DMSO d_6, 400 MHz).

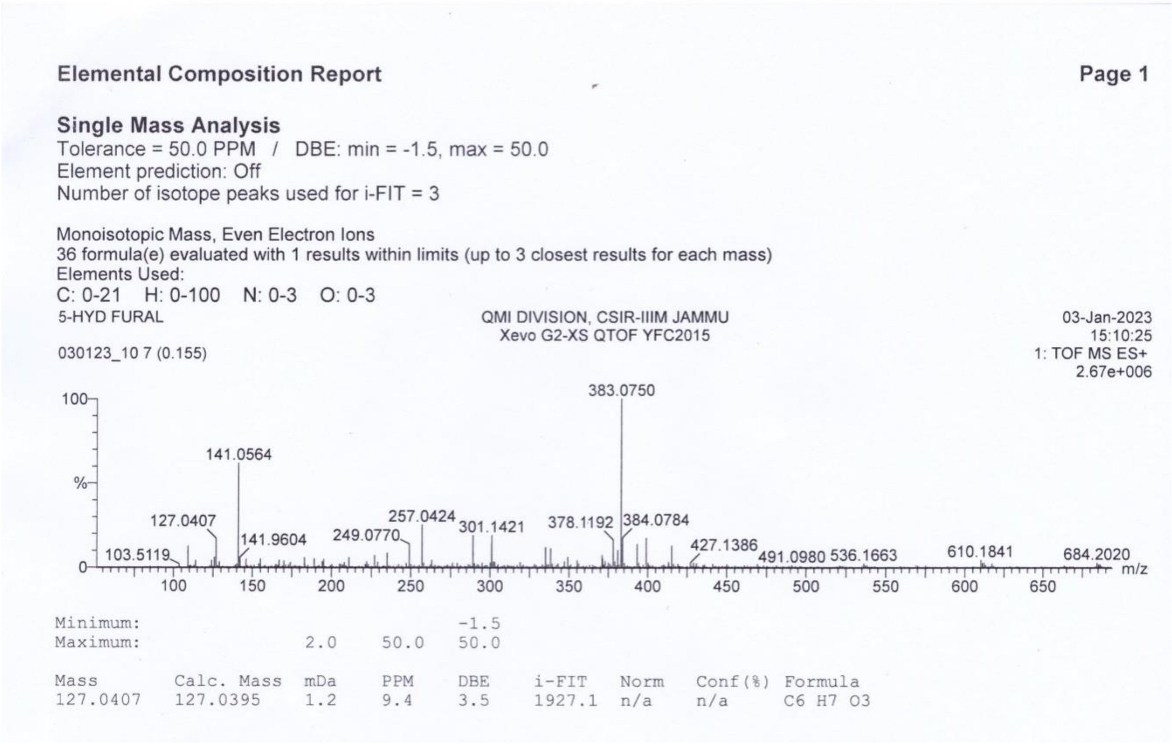

Figure S19: HRMS of 5-Hydroxymethyl-2-furaldehyde.

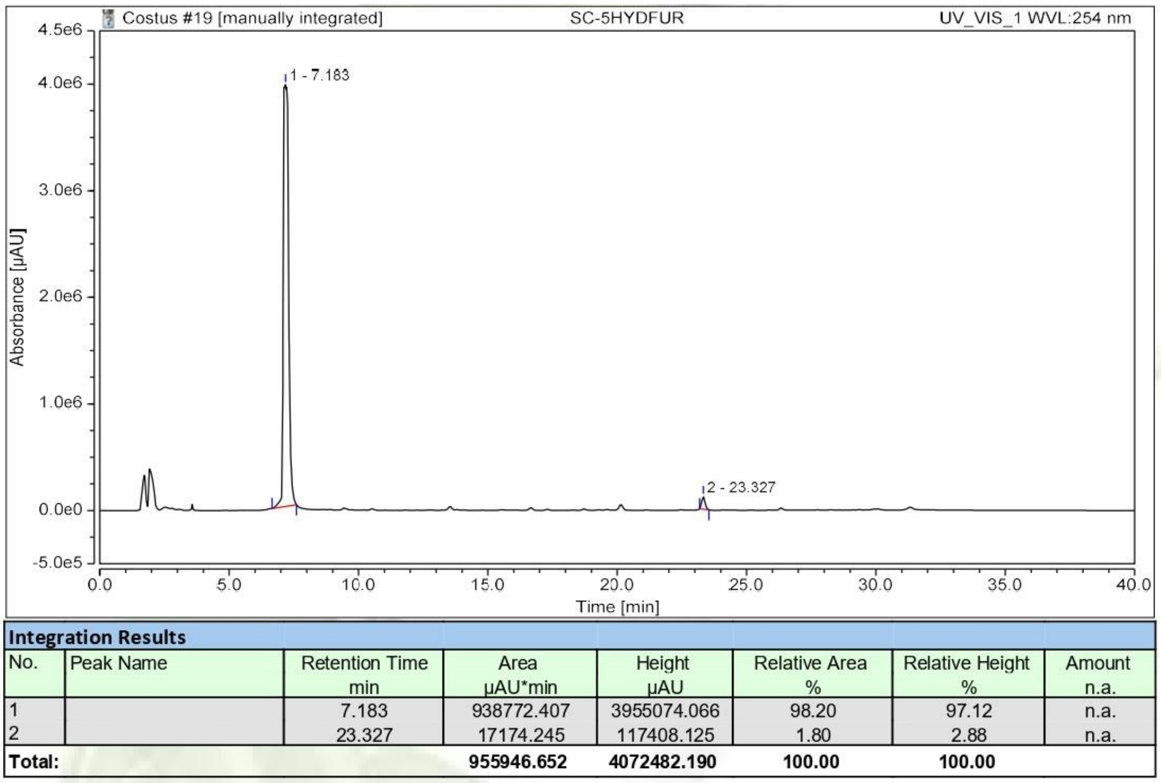

Integration Results

No.	Peak Name	Retention Time min	Area µAU*min	Height µAU	Relative Area %	Relative Height %	Amount n.a.
1		7.183	938772.407	3955074.066	98.20	97.12	n.a.
2		23.327	17174.245	117408.125	1.80	2.88	n.a.
**Total:**			**955946.652**	**4072482.190**	**100.00**	**100.00**	

Fig S20: Purity profile of compound 5-Hydroxymethyl-2-furaldehyde.

CALIBRATION PLOTS

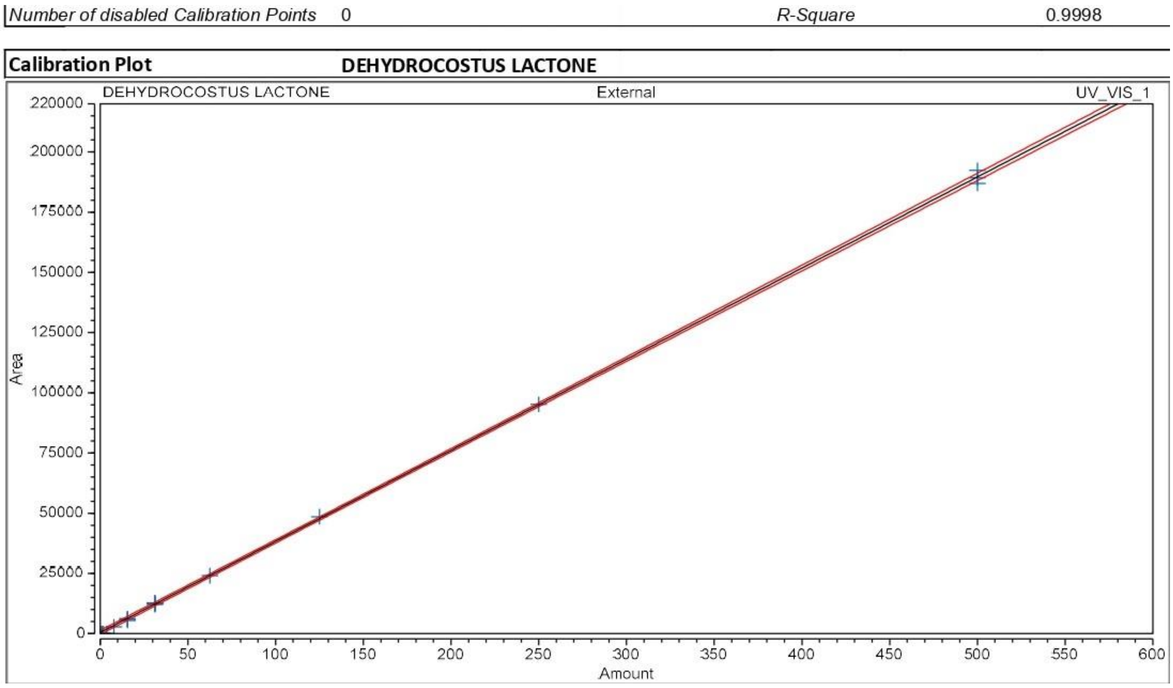

Fig S21: Calibration plot of compound Dehydrocostus lactone.

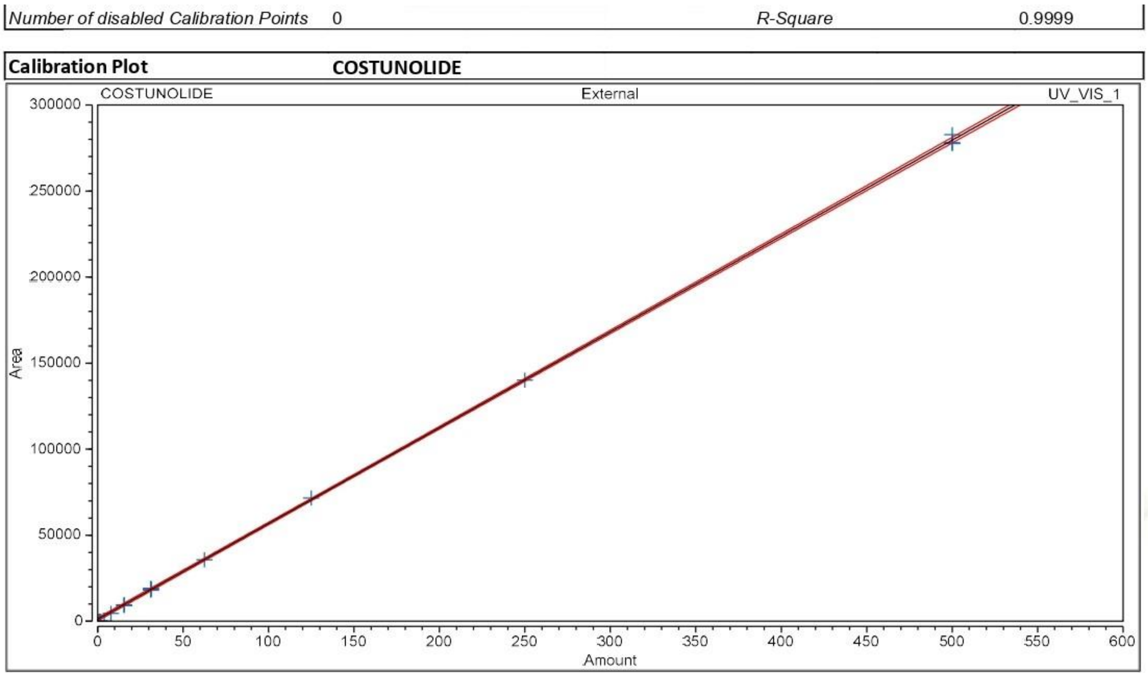

Fig S22: Calibration plot of compound Costunolide.

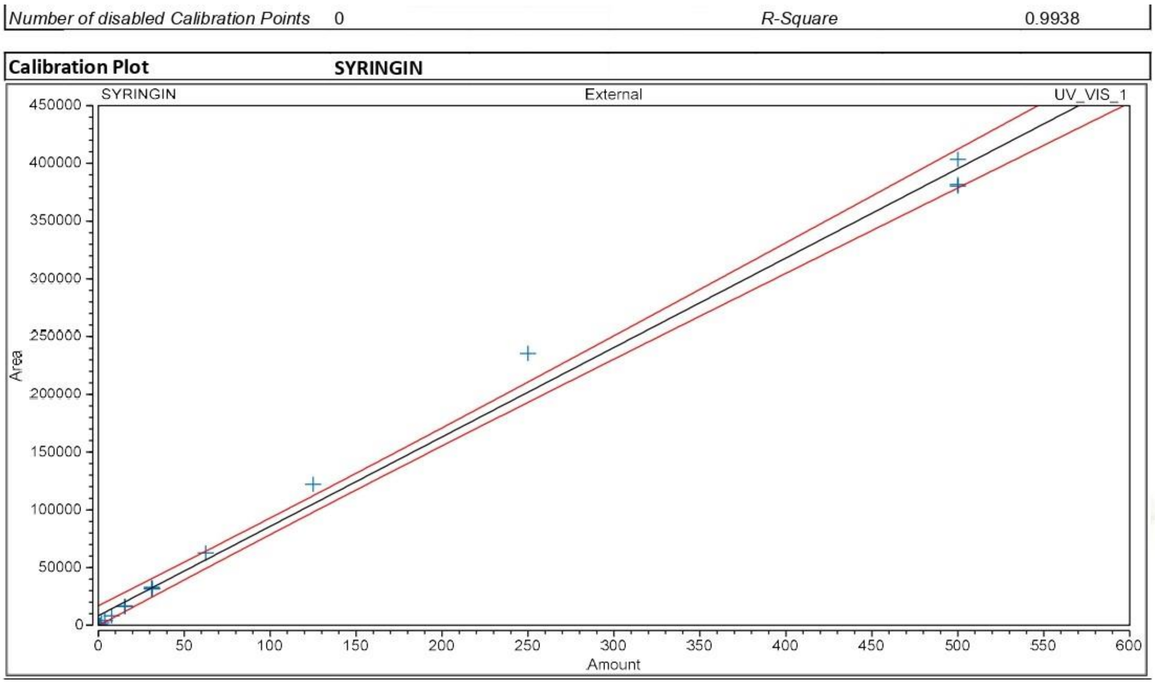

Fig S23: Calibration plot of compound Syringin.

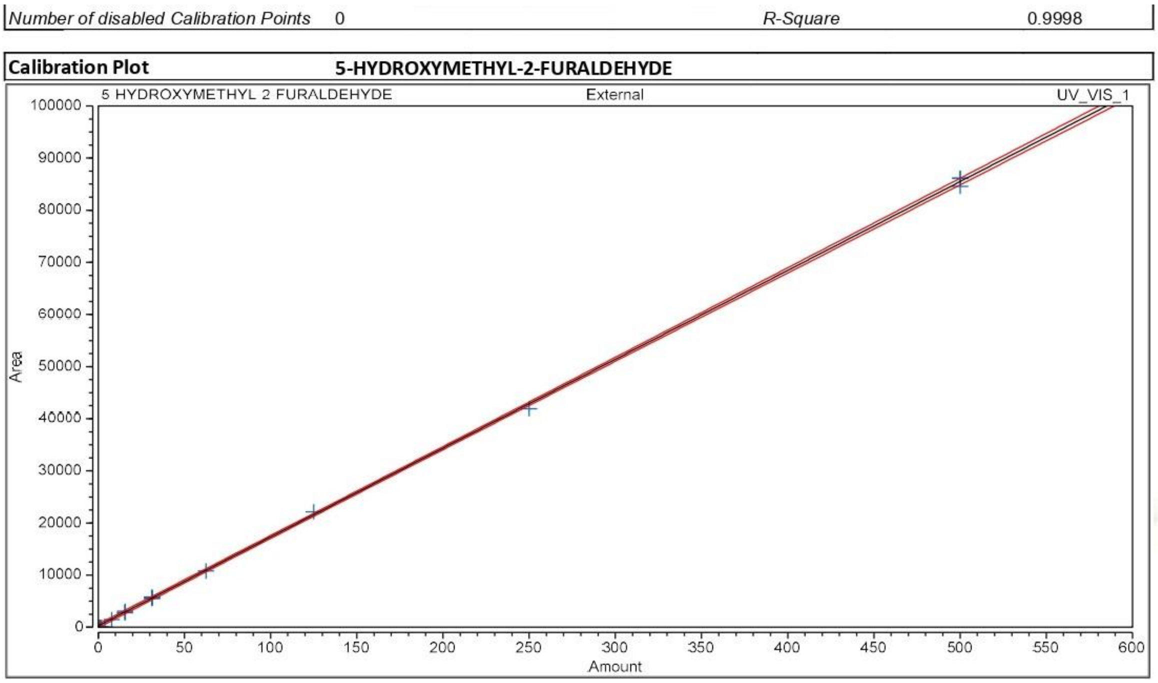

Fig S24: Calibration plot of compound 5-Hydroxymethyl-2-furaldehyde.

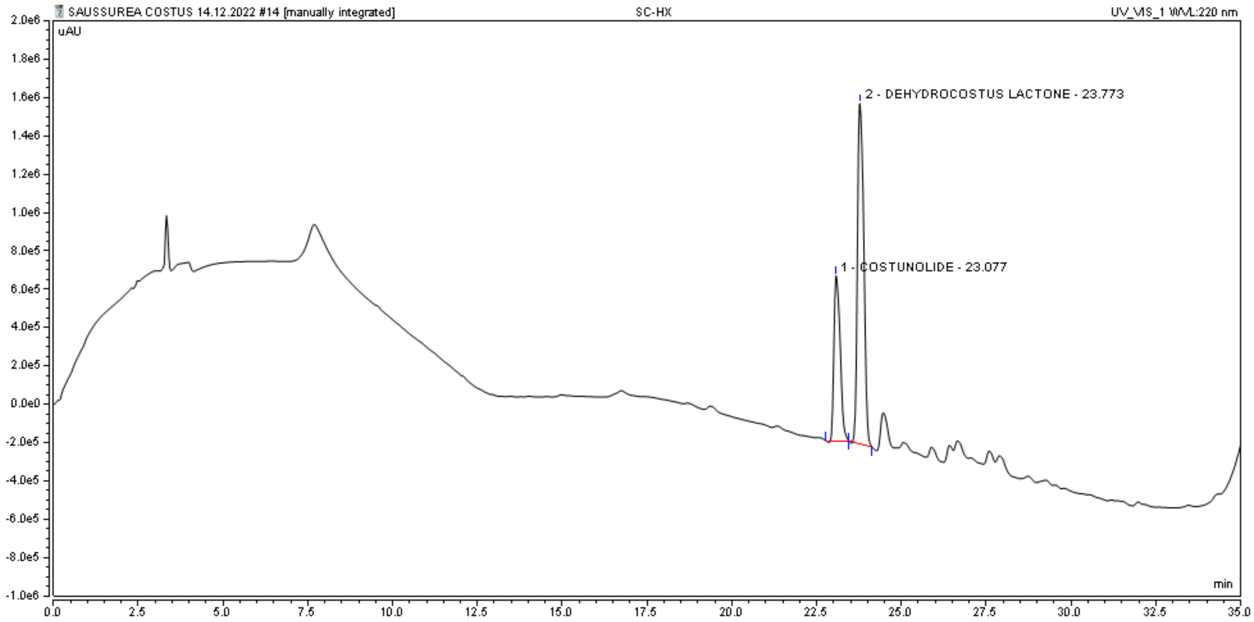

Fig S25: HPLC Chromatogram of Hexane fraction.

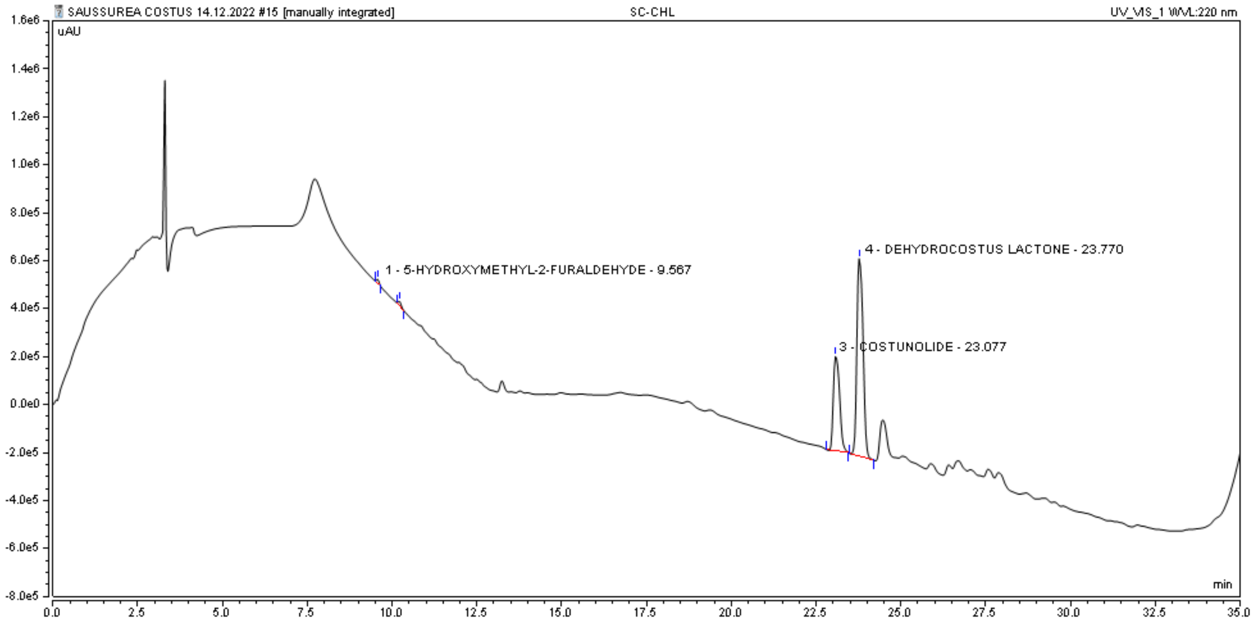

Fig S26: HPLC Chromatogram of Chloroform fraction.

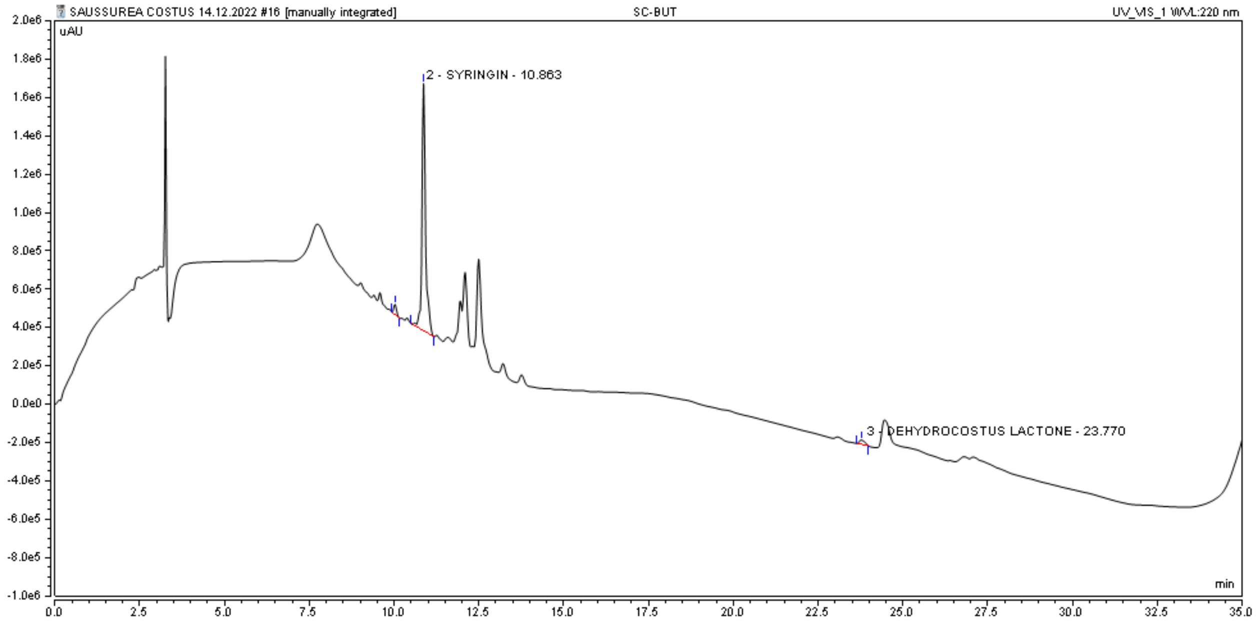

Fig S27: HPLC Chromatogram of Butanol fraction.
